# Fully Indium-Free Monolithic Two-Terminal Perovskite/Perovskite/Silicon Triple-Junction Solar Cells: Replacing All Four TCO Electrodes

**DOI:** 10.1021/acsenergylett.5c03919

**Published:** 2026-05-31

**Authors:** Maryamsadat Heydarian, Minasadat Heydarian, Sadaf Ghasemi, Andreas Fell, Oliver Fischer, Alexander J. Bett, Michael Günthel, Markus Knäbbeler-Buß, Florian Schindler, Martin C. Schubert, Juliane Borchert, Patricia S. C. Schulze, Stefan W. Glunz, Martin Bivour

**Affiliations:** † Fraunhofer Institute for Solar Energy Systems, Heidenhofstrasse 2, 79110 Freiburg, Germany; ‡ Chair for Photovoltaic Energy Conversion, Department of Sustainable Systems Engineering (INATECH), University of Freiburg, Emmy-Noether-Str. 2, 79110 Freiburg, Germany

## Abstract

Employing indium-based transparent conductive oxides (TCO) such as indium tin oxide (ITO) in silicon solar cells is already a hurdle due to the scarcity of indium for the photovoltaic community. This is even more challenging in perovskite/silicon multijunction solar cells where TCO layers are necessary for multiple applications beyond transparency and lateral carrier transport. Development of perovskite/perovskite/silicon triple-junction solar cells is a relatively new research focus. It is important to consider this criticality of the raw materials used during the design and development phase. In this work, we successfully replaced all four ITO layers used in our perovskite/perovskite/silicon triple-junction solar cell with an indium-free material. We mainly used zinc-doped tin oxide (ZTO), while for the silicon bottom cell’s front and rear contacts an interfacial layer of aluminum-doped zinc oxide (AZO) was used to ensure low contact resistance/efficient vertical transport to the silicon thin films. To replace the ITO top TCO of the perovskite top cell, a ZTO material with lower ZnO fraction was used to achieve a lower sheet resistance/more efficient lateral transport. With this, we present the first fully indium-free silicon-based triple-junction solar cell with no efficiency penalty.

## Graphical abstract

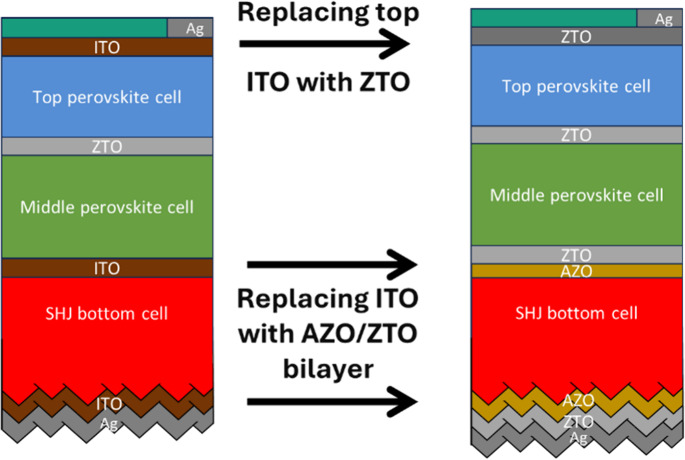

The perovskite-based triple-junction solar cell technology is a relatively new research field in the perovskite community. Triple-junction solar cells offer increased efficiency potential,^1^ and the lessons learned from the dual-junction cell development facilitates the advancement of this new technology. Since 2018 when the first proof of concept of a perovskite/perovskite/silicon triple-junction solar cell was reported by Werner et al.,^2^ to date the number of publications has significantly increased.^3–14^ The focus of these works has been on the development of the absorber layers, interconnection layers and compatible deposition techniques to achieve efficient solar cells. Besides the power conversion efficiency (*PCE*) improvement, which is the primary interest in triple-junction solar cells, so far upscaling and sustainability aspects of this new technology have received less attention. The current multijunction technology depends on indium-containing materials in the layer stack. It is already known that this is not suitable for mass production as the availability of indium is limited.^15^ Both perovskite/silicon tandem and triple-junction solar cells with silicon heterojunction (SHJ) bottom cell employ more than 200 nm indium-based TCO (mainly in silicon bottom cell). With this, indium consumption already reaches the annual global refinery of indium production (1080 t/a) by around 100 GWp of installed capacity.^16^ It is therefore crucial to minimize the use of indium to allow for scaling up to a terawatt production scale. We have recently shown that the indium tin oxide (ITO) recombination layer between two perovskite subcells (ii: rec TCO_top/mid_ in Figure 1) can be replaced by zinc tin oxide (ZTO) without sacrificing the efficiency.^16^ Motivated by this achievement and considering the criticality of the indium material, we aim to substitute the other three ITO layers as well, i.e., the top TCO electrode on top of the perovskite top cells (i: top TCO in Figure 1), the recombination layer between the silicon bottom cell and perovskite middle cell (iii: TCO_mid/bot_) and the rear TCO of the silicon bottom solar cell (iv: rear TCO). However, this is not a trivial task, as the requirements for each layer differ depending on its position and function in the cell structure. For example, all four TCOs must (a) provide light incoupling for the relevant spectral range of the respective absorber (indicated in Figure 1), ensure good (b) energy alignment/good vertical transport with the adjacent layers (ETL, HTL, metal) and (c) can influence the growth of subsequent films.^17,18^ For the top TCO (d), lateral transport, i.e., low sheet resistance, is another requirement. (e) When solvents are involved in the processing of perovskite, a solvent barrier function could be one concern for the recombination layer between two perovskite subcells. However, mostly other layers in the structure such as the buffer layer serve as the solvent barrier layer. A barrier function (for example, against humidity) of the top TCO as the outer layer of the device might also be relevant. In addition, the process temperature limitation differs if the layer is processed on the SHJ bottom cell ( ∼ 200 °C) or on perovskite subcells ( ∼ 100 °C).

**Figure 1:**
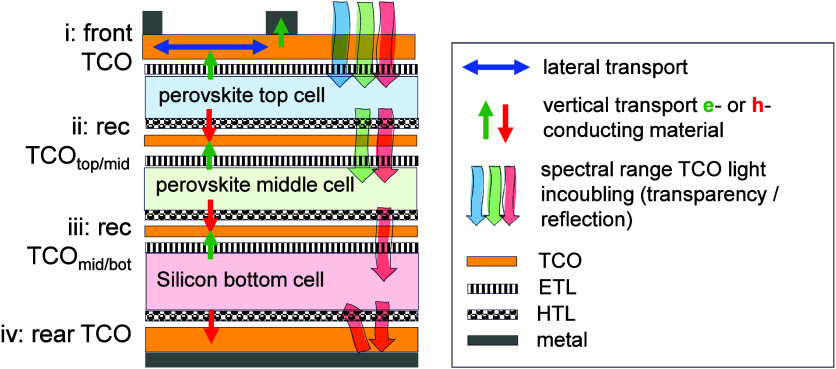
Simplified representation of the role of the four TCO layers (top TCO: ∼25 nm, TCO_top/mid_: ∼15 nm, TCO_mid/bot_: ∼20 nm and rear TCO: ∼190 nm) in the perovskite/perovskite/silicon triple-junction solar cell.

In this work, we first implement zinc doped tin oxide (ZTO) as the front and rear TCOs of the silicon bottom cell, using the recently developed TCO_top/mid_ recombination layer.^16^ We then apply a slightly modified ZTO material to implement it as the top TCO of the final triple-junction device. This is the first demonstration of a fully indium-free silicon bottom cell based on ZTO and the implementation of ZTO as a top TCO in multijunction solar cells. With these learnings from the subsequent replacement of the TCO electrodes, we report on the first all-indium-free triple-junction solar cells with performance similar to the ITO-based baseline. The champion fully indium-free monolithic two-terminal perovskite/perovskite/silicon triple-junction solar cell features an open circuit voltage (*V*_OC_) of 3.1 V, which is among the highest values reported for this structure, a fill factor (*FF*) of 82%, a short-circuit current density (*j*_SC_) of 9.0 mA/cm^2^, and a power conversion efficiency (*PCE*) of 22.6%.

The bottom cell in this work is a flat front and rear textured SHJ solar cell as shown in Figure 2a. Twenty nm of ITO serve as the recombination layer between middle perovskite and the silicon cell and 190 nm of ITO is deposited at the rear which is necessary to facilitate the charge transport as well as enhance the near-infrared reflection.^19^ The recombination layer thickness has been optimized in a previous study.^20^ Replacement of these two layers is absolutely crucial, as in particular the rear TCO accounts for the largest indium consumption in our devices.^16^ It should be noted that, for practical reasons, we used monofacial silicon bottom cells with full area rear-side metallization, where lateral transport in the TCO at the rear is not relevant. We still employ a thicker rear TCO of about 190 nm for optical reasons.^19^ This differs from the industrial bifacial silicon cell design, which employs a metal grid electrode on both the front and rear side^21^ and only 100 nm of TCO at the rear.

**Figure 2:**
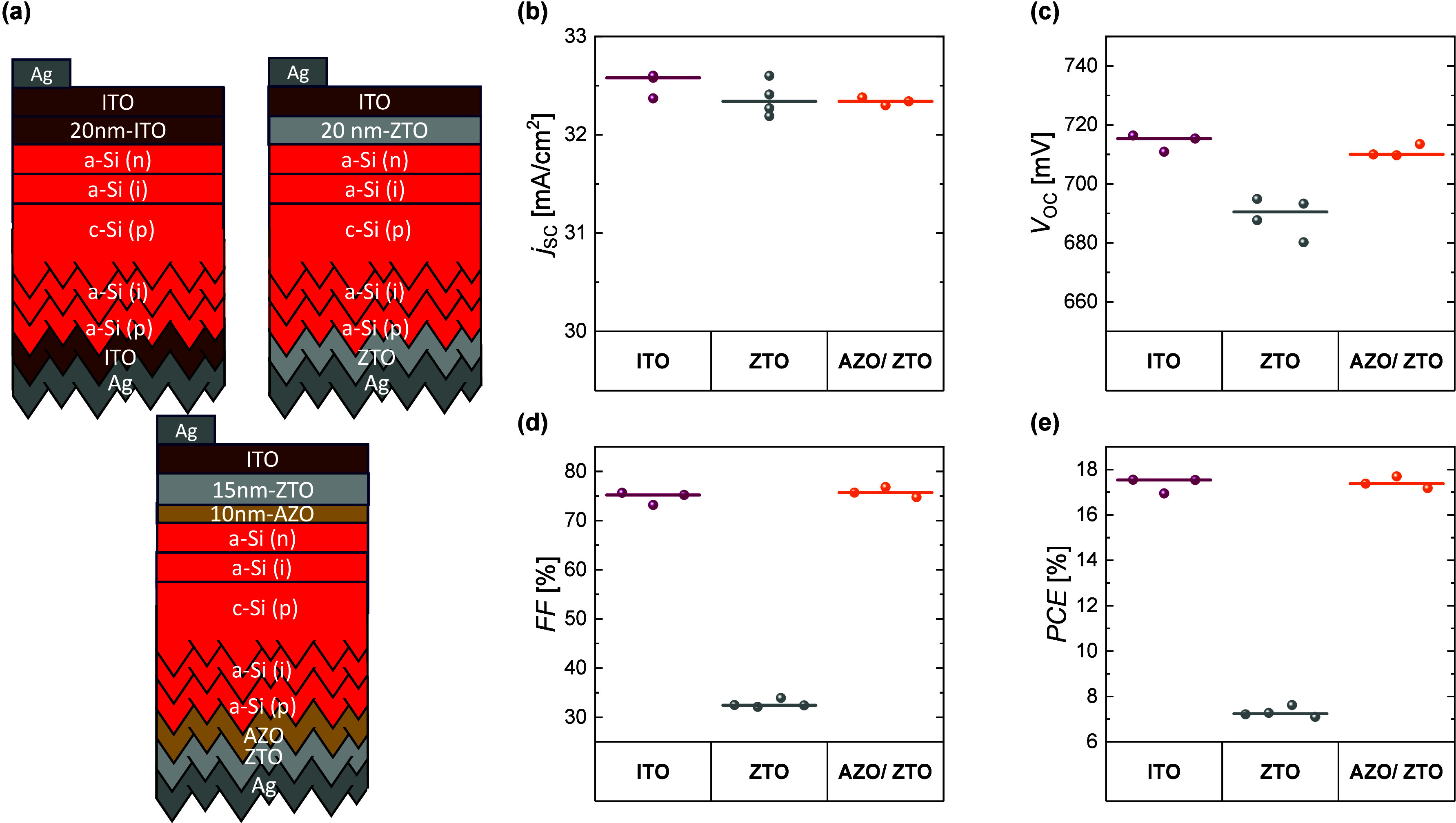
(a) Schematic of the silicon heterojunction solar cells and (b–e) the photovoltaic parameters of silicon single-junction devices with ITO, ZTO and AZO/ZTO as front and back TCO.

Due to the complexity of the device fabrication and interpretation of the data, we first studied the performance using single-junction silicon bottom cells. This allows us to evaluate the effect of the ITO replacement independent of the effects of the perovskite subcells. For this purpose, the front electrode was completed with an additional 50 nm ITO layer to enable sufficient lateral transport and a silver grid. Figure S1 in Supporting Information presents the photovoltaic (PV) parameters of the single-junction SHJ test solar cells. Upon replacement of the rear ITO with ZTO there is more than 17% absolute drop in the *FF* of the devices. This is even more significant when only front ITO is replaced, which can be attributed to increased series resistance (Figure S2 in Supporting Information). Consequently, the SHJ bottom cells with front and rear ZTO layers do not perform on the same level as the ITO-based solar cells. When a 10 nm ITO is deposited prior to the rear ZTO, the *FF* improved by 20% absolute compared to the solar cells with only ZTO. Hence, the issue originates from the poor contact of the ZTO layer with lowly doped amorphous silicon (a-Si) as reported elsewhere.^22,23^ To address this challenge while aiming to create indium-free solar cells, we sputtered 10 nm of aluminum doped zinc oxide (AZO), which has demonstrated good contact formation with amorphous silicon.^24^ Figure 2 compares the PV parameters of solar cells with bilayers of (AZO/ZTO), ZTO and ITO as front and rear TCO. By adding the AZO layer prior to the ZTO deposition, the *FF* of the indium-free solar cells is maintained at the same level as for the reference group with similar series resistant (Figure S4). Given the poor carrier density of the ZTO, it is likely that poor tunneling governs the high contact resistivity compared to the AZO with higher carrier density.^25^ When the bilayer of AZO/ZTO is used, the effective carrier density is higher and close to the AZO layer, as shown in Table S4. It is important to note that in state of the art SHJ solar cells with nanocrystalline electron and hole contacts instead of a-Si layers^26^ or TOPCon based passivation contacts, the contact resistivity with such lowly doped TCO is not a problem as shown in ref 23, and therefore, in such cases, no additional AZO layer is needed.

The photovoltaic parameters of the devices before and after annealing are presented in Figure S3. We note that the deposition of ZTO and AZO was done with the substrate at room temperature and that annealing for 5 min at 180 °C was applied to improve *FF* and *V*_OC_. However, such annealing was not beneficial in case of silicon solar cells with only ZTO layers. Upon annealing, there is an improvement of ∼20 mV in *V*_OC_ and ∼11% improvement in *FF* for the samples with AZO/ZTO. The improvement is much less for the ITO-based reference solar cells (only ∼4 mV in *V*_OC_ and less than 2% in *FF*), most likely caused by the fact that ITO deposition is already done at ∼180 °C resulting in in situ annealing. In addition, silicon solar cells with only AZO as front and back TCO were also fabricated. Their *V*_OC_ and *FF* are on the same level with AZO/ZTO based devices (Figure S3). Their performance is slightly lower due to lower *j*_SC_ resulting from the higher carrier density of AZO. However, the main motivation for using ZTO especially on the front was the poor formation of our wet chemically processed SAM/perovskite on the surface of AZO, as shown in Figure S5. We note that no significant sputter-induced damage is introduced by the tested TCOs (ITO, AZO, and ZTO), as evidenced by comparable implied open-circuit voltage (i*V*_OC_) imaging done on the glass/perovskite/C_60_/SnO_*x*_/TCO stack (Figure S7).

Perovskite/perovskite/silicon triple-junction solar cells were then fabricated on the optimized indium-free SHJ bottom cells using AZO/ZTO as the rear TCO and as the recombination layer between silicon and perovskite subcells. The structure of the device is therefore SHJ/AZO/ZTO/poly-[bis­(4-phenyl)-(2,4,6-trimethylphenyl)-amin]­(PTAA)/poly­[(9,9-bis­(3′-(*N*,*N*-dimethylamino)­propyl)-2,7-fluorene)-*alt*-2,7-(9,9-dioctylfluorene) bromide]­(PFN-Br)/middle bandgap (MBG) perovskite/C_60_/SnO_*x*_/ZTO/2PACz/high bandgap (HBG) perovskite/C_60_/SnO_*x*_/ITO/Ag/MgF_2_, as shown in Figure 3a. The MBG and HBG perovskite compositions are Cs_0.05_(FA_0.90_MA_0.10_)_0.95_Pb­(I_0.95_Br_0.05_)_3_ and Cs_0.20_FA_0.71_MA_0.09_Pb­(I_0.64_Br_0.27_Cl_0.09_)_3_, respectively. The bandgaps determined from the inflection point of the EQE (dEQE/dE), are 1.85 eV for the top perovskite subcell and 1.57 eV for the middle perovskite subcell (Figure 3c). These cells feature an indium-free bottom cell, and an indium-free recombination layer between perovskite subcells as demonstrated in our previous work.^16^ At this point, only the front ITO still contains indium and will be replaced in the next step.

**Figure 3:**
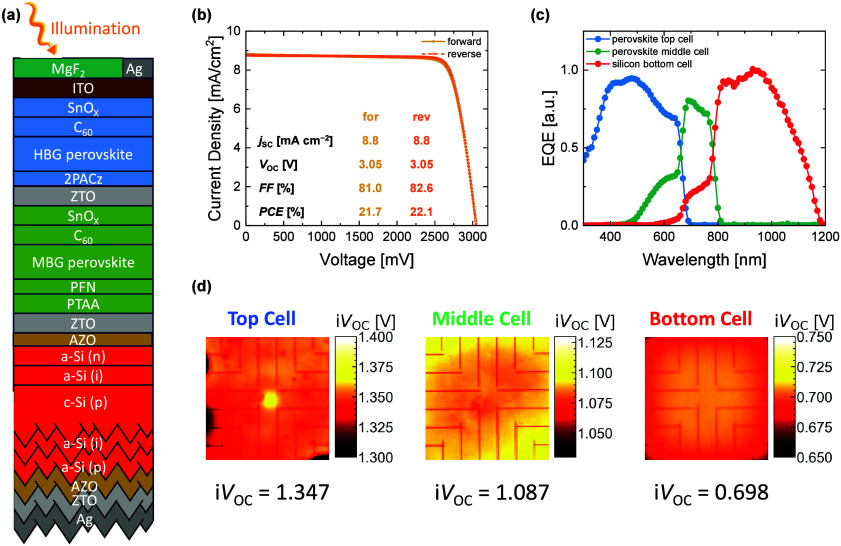
(a) Schematic of the perovskite/perovskite/silicon triple-junction solar cell structure. The illumination side is shown with the yellow arrow. (b) *jV* curves, (c) EQE curves, and (d) subcell selective i*V*_OC_ images of triple-junction solar cell with an AZO/ZTO-based bottom cell.

The triple-junction solar cell on this indium-free bottom cell shows an excellent *V*_OC_ value of 3.05 V (Figure 3b). i*V*_OC_ images of the individual subcells are shown in Figure 3d. The bottom cell has an i*V*_OC_ of around 0.7 V, which is similar to the i*V*_OC_ of ITO-based silicon bottom cells^14^ and is an indication that the new TCO does not impair the passivation quality.^28^ A *V*_OC_ of 3.09 V was determined for this sample after i*V*_OC_ characterization (the difference to the *V*_OC_ of 3.05 V from the *jV* measurement can be explained by a difference in the applied illumination intensity. The illumination of the middle solar cell for the i*V*_OC_ measurements is likely higher compared to the calibrated spectrum during the *jV* measurements). The selectivity loss (i.e., i*V*_OC_ – *V*_OC_) is less than 50 mV, indicating that the cell has the potential for even higher *V*_OC_.

After successfully replacing the mentioned ITO layers, we addressed the remaining one in the solar cell structure, namely, the top TCO. One of the challenges of using the same ZTO layer for the top TCO is the higher sheet resistance compared to our standard ITO which limits the lateral conductivity needed. The top ITO used as reference in this work is a 25 nm sputtered ITO developed by Kabakli et al.^29^ The sheet resistance of the ZTO layer with approximately the same thickness is around 7800 Ω/□, much higher than 189 Ω/□ measured for ITO (Figure 4a). The sheet resistance can be reduced by either increasing the thickness of the ZTO layer or, as we found out by adapting the ZTO target composition, more precisely by reducing the fraction of the ZnO doping. The new target has a nominal composition of (SnO_2_/ZnO = 99/1 wt %) compares to the initial target which has a nominal composition of (SnO_2_/ZnO = 92/8 wt %). The composition of the sputtered film was analyzed using X-ray Photoelectron Spectroscopy (XPS) measurement. As expected, the film from the SnO_2_/ZnO = 99/1 wt % target has a Sn_0.992_Zn_0.008_O_1.396_, and the film from the SnO_2_/ZnO = 92/8 wt % has a Sn_0.910_Zn_0.087_O_1.475_ composition. Detail of the elemental composition can be found in Table S5. The composition of the sputtered ZTO has shown to have a direct impact on its electrical properties.^30^ To analyze such effects, Hall measurement was performed on the 100 nm thick ZTO films (Table 1); the ZTO with reduced zinc doping, which we name ZTO (99/1), hereafter, shows higher carrier concentration and mobility. As a result, the ZTO with reduced ZnO concentration shows much lower resistance for the same thickness.

**Table 1:** Electrical Properties of the Two ZTO Films Extracted from Hall Measurements, Including Hall Mobility (μ), Hall Carrier Concentration (N*), and Mean Sheet Resistance (*R*)

film	thickness (nm)	μ [cm^2^/(V s)]	N* [×10^20^ cm^–3^]	*R* [ohm]
ZTO (92/8)	100	12.7	0.3	1570
ZTO (99/1)	100	19.2	0.8	490

**Figure 4:**
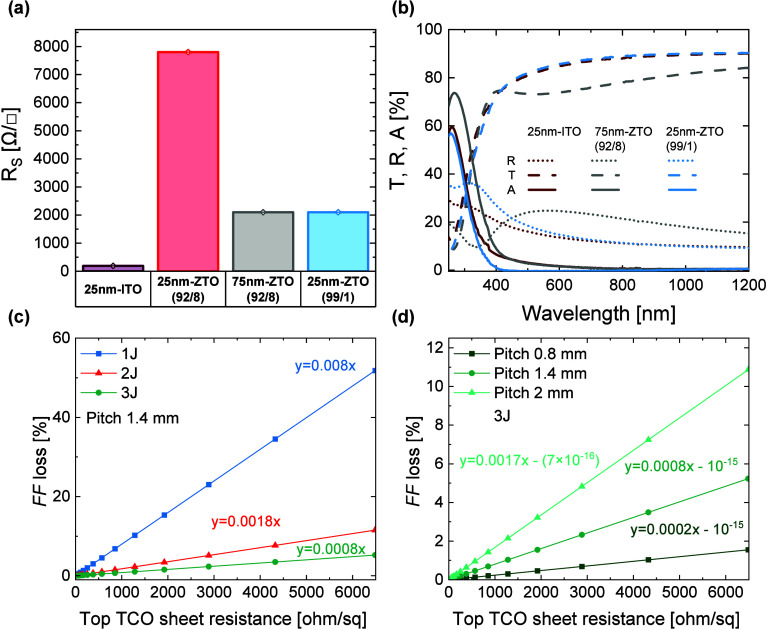
(a) Measured sheet resistance and (b) transmittance, reflectance, and absorptance spectra of ITO and ZTO layers deposited on glass substrate. (c) Modeled *FF* loss from resistive power losses as a function of top-TCO sheet resistance for single-junction (1J), dual-junction (2J), and triple-junction (3J) solar cells assuming a grid finger of 1.4 mm, and (d) a modeled *FF* loss from resistive power losses of the TCO/grid electrode as a function of top-TCO sheet resistance for a grid finger spacing of 0.8, 1.4, and 2 mm for triple-junction solar cells.

For top TCO application we tested both thicker ZTO (92/8) with 75 nm and the new ZTO (99/1) with 25 nm which show an *R*_s_ of approximately 2100 Ω/□ (determined from a four-point probe measurement; Figure 4a), respectively. To have a fair comparison, similar sputtering parameters were used for deposition of both ZTO layers as properties of sputtered metal oxide heavily depend on the deposition parameters such as reactive gases present during the sputtering.^30,31^ We note that the oxygen flow rate used led to the minimum sheet resistance, as shown in Figure S8.

Further, optical properties of the ZTO and ITO layers were compared. Figure 4b shows the optical properties (transmittance, reflectance, and absorptance) of the 25 nm ITO reference, 75 nm ZTO (92/8), and 25 nm ZTO (99/1). The thicker ZTO layer shows higher reflection and consequently lower transmission. However, the reflection and transmission in the final device might vary since the surrounding layers are different. The ZTO layer with lower zinc concentration exhibits similar transparency as compared to the reference ITO layer. In addition, the properties of the ZTO (92/8) and ZTO (99/1) films are compared to a reference 25 nm sputtered ITO. The X-ray diffraction (XRD) analysis shows no diffraction peaks, confirming that all three TCOs have an amorphous structure (Figure S9). Please note that the measurements were performed on as-deposited films with no annealing.

*FF* losses of single-junction, dual-junction and triple-junction solar cells were modeled using the solar cell simulation software Quokka3 ^32^ to analyze the losses arising from the higher sheet resistance of top TCO. Even though the sheet resistance of the ZTO is still higher than that of the reference ITO, this only has a moderate influence on the transport losses since the solar cell has a low current density. Hence in two-terminal multijunction solar cells, the higher the number of junctions the lower the current, and therefore less ohmic transport loss is expected for a given resistance as shown in Figure 4c. However, even in the case of a more resistive TCO layer, by adjusting the metallization such that a narrower grid finger spacing is used, the transport loss becomes even less prominent (Figure 4d), at the cost of increased shading losses. Since we use metallization with 1.4 mm spacing between the grid fingers, using a top TCO layer with a sheet resistance of 2100 Ω/□ in our triple-junction device, we do not expect a significant *FF* loss. In addition to the transport properties, the *FF* of a multijunction solar cell depends on the current matching between its subcells.^33^ Therefore, optical interference and different reflection properties, e.g., as a result from different top TCO thickness, can have an indirect impact on the *FF* of solar cells as well.

We then fabricated triple-junction solar cells with the structure SHJ/AZO/ZTO (92/8)/2PACz/MBG perovskite/C_60_/SnO_*x*_/ZTO/2PACz/HBG perovskite/C_60_/SnO_*x*_/top TCO/Ag/MgF_2._ The top TCO is either 75 nm of ZTO (SnO_2_/ZnO = 92/8 wt %) or 25 nm of ZTO (SnO_2_/ZnO = 99/1 wt %) and compared it with our standard 25 nm ITO. The photovoltaic parameters of these three groups are shown in Figure 5. When replacing the standard ITO with 75 nm of ZTO (92/8), *j*_SC_ drops by 0.4 mA/cm^2^ which results in lower *PCE* of this group. When the ZTO with a lower Zn fraction (99/1) is used, *j*_SC_ is within the same range as ITO-based devices. Reflection spectra of this group also match well with devices using ITO as the top TCO and higher reflection for the 75 nm TCO is seen in the wavelength range relevant for the perovskite subcells (see Figure S10). In addition, the *FF* of the devices with ZTO (99/1) is at the same level as that for the ITO group. Therefore, the performance of the samples is within the same range as the reference group since there is no significant drop in any of the photovoltaic parameters. The champion solar cells from all groups are presented in Figure S11 in the Supporting Information. For the ITO-based device, a *PCE* of 22.9%, with *V*_OC_ of 3083 mV, a *FF* of 81.9%, and a *j*_SC_ of 9.07 mA/cm^2^ is achieved while the device with 25 nm of ZTO (99/1) shows a *PCE* of 22.6%, with *V*_OC_ of 3074 mV, a *FF* of 81.8%, and a *j*_SC_ of 8.98 mA/cm^2^. To gain some insight into higher transport and *FF* losses originating from the higher sheet resistance of the ZTO layer we again used silicon single-junction test cells (Figure S12). 75 nm ITO, 75 nm ZTO (92/8), and 25 nm ZTO (99/1) as top TCO were compared. As expected from the higher sheet resistance (Figure 4a), devices with the ZTOs show a reduction in *FF* accompanied by increased series resistance. In addition, the two TCO thicknesses exhibit distinct optical properties, which are also evident from the different colors of the final devices (Figure S12). Finally, for initial stability evaluation of the indium free devices, an unencapsulated triple-junction solar cell was stressed at a voltage close to maximum power point in ambient conditions (25 °C, ≈30% relative humidity) under 1-sun illumination for a longer time. Figure S13 shows that ∼99% of the power output was maintained for more than 6 h of measurement time.

**Figure 5:**
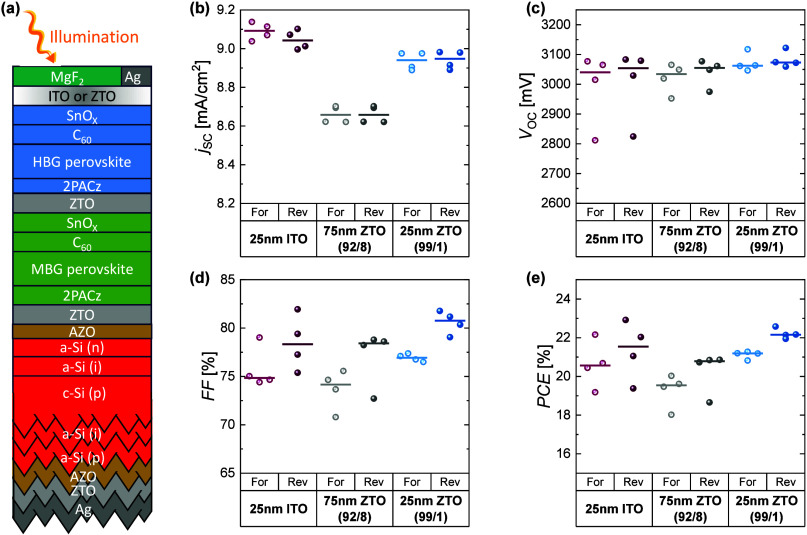
(a) Schematic of the perovskite/perovskite/silicon triple-junction solar cell structure. The illumination direction is shown with the yellow arrow. (b–e) The photovoltaic parameters comparing ITO to ZTO as the top TCO.

In summary, we successfully replaced all ITO layers in monolithic two-terminal perovskite/perovskite/silicon triple-junction solar cells. First, an indium-free SHJ solar cell was developed by using an AZO/ZTO bilayer as the top and rear TCO. Since the contact resistivity between ZTO and a-Si is high, the addition of a thin layer of AZO was required. Second, by reducing the ZnO fraction from 8% to 1% in our rotary ZTO target using DC sputtering in an industry-like in-line sputter tool, the sheet resistance of 25 nm low temperature ZTO was decreased by 5700 Ω/□. Implementing the developed ZTO as top TCO, a fully indium-free triple-junction solar cell with voltage (*V*_OC_) of 3074 mV, a fill factor (*FF*) of 81.8%, a short-circuit current density (*j*_SC_) of 8.98 mA/cm^2^, and a power conversion efficiency (*PCE*) of 22.6% was achieved, which is on similar level as the ITO-based solar cell. This important achievement paves the way toward indium-free multijunction solar cells. The current sheet resistance of the developed ZTO is suitable to be used as a top TCO in triple-junction solar cells. For implementation of the developed ZTO as top TCO of the single and dual-junction solar cell, to avoid transport loss, the sheet resistance may need to be further reduced, for example, by increasing the thickness of ZTO and its carrier density and mobility. Even though the tin consumption increases as the target material has a significantly higher tin content, it does not impose any shortage as shown in.^16^ In addition, since ZTO is a lower-cost material compared to ITO, this approach would still be cost-effective.

## Supplementary Material



## Notes

Detail on solar cell fabrication; *EQE* measurements; *IV* measurements; Reflection measurements; UV–vis measurements; i*V*_OC_ imaging; XPS measurement; Solar cell modeling; Additional experimental data on silicon single-junction solar cells and perovskite/perovskite/silicon triple-junction solar cells and TCO films (PDF)
