# Supplementary material for: Fully Indium-Free Monolithic Two-Terminal Perovskite/Perovskite/Silicon Triple-Junction Solar Cells: Replacing All Four TCO Electrodes
Source: ACS Energy Lett. 2026 May 31;11(6):4308–15. doi: 10.1021/acsenergylett.5c03919 (PMC13274487; doi:10.1021/acsenergylett.5c03919)
Supplement: Supplementary file 1 [file nz5c03919_si_001.pdf]

# Fully Indium-Free Monolithic Two-Terminal Perovskite/Perovskite/Silicon Triple-Junction Solar Cells: Replacing All Four TCO Electrodes

*Maryamsadat Heydarian*[1], Minasadat Heydarian[1,2], Sadaf Ghasemi[1], Andreas Fell[1], Oliver Fischer[1], Alexander J. Bett[1], Michael Günthel[1], Markus Knäbbeler-Buß[1], Florian Schindler[1], Martin C. Schubert[1], Juliane Borchert[1,2], Patricia S. C. Schulze[1], Stefan W. Glunz[1,2], Martin Bivour[1]*

[1] Fraunhofer Institute for Solar Energy Systems, Heidenhofstrasse 2, 79110 Freiburg, Germany

[2] Chair for Photovoltaic Energy Conversion, Department of Sustainable Systems Engineering (INATECH), University of Freiburg, Emmy-Noether-Str. 2, 79110 Freiburg, Germany

AUTHOR INFORMATION

**Corresponding Author**

* *Maryamsadat Heydarian Email: Maryamsadat.Heydarian@ise.fraunhofer.de*

## Material and method

*Material and solutions preparation*

Lead iodide ($PbI_2$), lead bromide ($PbBr_2$) and lead chloride ($PbCl_2$) powders are purchased from TCI. Methylammonium bromide (MABr) and formamidinium iodide (FAI) powders from GreatCell Solar. Cesium iodide (CsI), methylammonium chloride (MACl), PFN-Br (poly(9,9-bis (3'-(*N,N*-dimethyl)-*N*-ethylammoinium-propyl-2,7-fluorene)-alt-2,7-(9,9-dioctylfluorene)) dibromide), PTAA (poly[bis(4-phenyl)(2,4,6-trimethylphenyl)amine]) from Sigma Aldrich. 2PACz ([2-(9H-carbazol-9-yl) ethyl] phosphonic acid) and piperazinium iodide (PI) are from Dyenamo.

The middle bandgap perovskite has a $Cs_{0.05}(FA_{0.90}MA_{0.10})_{0.95}Pb(I_{0.95}Br_{0.05})_3$ composition and the high bandgap perovskite has a $Cs_{0.20}FA_{0.71}MA_{0.09}Pb(I_{0.64}Br_{0.27}Cl_{0.09})_3$ composition. The perovskite solutions were prepared according to our previous work [1].

*Silicon solar cell fabrication*

Silicon solar cells in this work were fabricated using 250 µm-thick p-type float-zone silicon wafers (bright etched, Siltronic base resistivity of about 1 Ω cm). Potassium hydroxide (KOH) was used to etch a pyramidal texture onto the rear side only using an industrial tool (SINGULUS SILEX). Afterwards the silicon oxide used to protect the planar front side during texturing was etched off. The wafers were then subjected to $O_3$-based wet-chemical cleaning and transferred to the plasma-enhanced chemical vapor deposition (PECVD) cluster tool (INDEOtec Octopus II). A stack of intrinsic/doped amorphous silicon passivation layers (~19 and 27 nm for the planar front and textured rear surface, respectively) was deposited on both sides. The PECVD process was carried

out in a parallel-plate reactor, with 13.56 MHz power and a temperature of 200 °C, using a mixture of silane ($SiH_4$), hydrogen ($H_2$), phosphine ($PH_3$), and trimethylborane (TMB).

For the devices with ITO, a ~ 20 nm recombination layer was formed through a 1 $cm^2$ shadow mask on the front via direct-current (DC) sputtered ITO ($In_2O_3/SnO_2$ = 97/3 wt %) in an VON ARDENNE SCALA in-line tool using a mixture of oxygen and argon (oxygen/argon 5.3%) and intentional heating resulting in a sample temperature of ~180 °C. The rear contact was completed by ~190 nm of ITO. For the devices with ZTO, 20 nm of ZTO was sputtered using VISTARIS 600 in-line tool from SINGULUS without heating utilizing the same shadow mask on the front. On the rear side, 190 nm of ZTO was deposited with no mask. The ZTO rotary target was provided and co-developed with GfE. For samples with AZO/ZTO bilayer, prior to the deposition of 15 nm ZTO, 10 nm of AZO ($ZnO:Al_2O_3$ 99:1 wt %) was sputtered. On the rear side ~1000 nm of Ag was deposited on the full area. Finally, the wafers were laser-scribed to 2.5 × 2.5 $cm^2$ substrates, with the 1 $cm^2$ TCO pad in the middle defining the active area.

For the single-junction SHJ solar cells, 50 nm ITO ($In_2O_3/SnO_2$ = 90/10 wt %, no heating) was sputtered with an Oxford cluster tool through a 1 $cm^2$ shadow mask and for the busbar and grids, 500 nm Ag was deposited on top to finish the solar cells.

*Triple-junction solar cell fabrication*

The silicon solar cells were UV-Ozone treated for 15 minutes. For the samples with PTAA, 120 µL PTAA was spin coated at 6000 rpm for 30 s and annealed on a 100 °C pre-heated hot plate for 10 min in the $N_2$ filled glovebox. 60 µL PFN was then spin coated on top of PTAA for 20 s at 5000 rpm to improve the wettability. For the samples with 2PACz, 130 µL of 2PACz solution were spin coated for 30 s at 3000 rpm followed by annealing for 10 min at 100 °C. The middle

bandgap perovskite solution (150 μL) was spin coated at 4000 rpm for 35 s with a ramp of 3 s. 300 μL ethyl acetate was dropped on the spinning substrates after 10 s. Samples were annealed for 30 min at 100 °C. Afterwards the $C_{60}$ layer 15 nm was thermally evaporated with an MBraun evaporation tool with 0.2 Å/s evaporation rate. 30 nm $SnO_X$ layers were deposited by atomic layer deposition ALD (182 cycles). 15 nm ZTO ($SnO_2$/ZnO = 92 /8 wt%) was sputtered with VISTARIS 600 tool through a shadow mask that defines 1 $cm^2$ cell active area. Afterward, 130 μL of 2PACz solution was spin coated for 30 s at 3000 rpm followed by annealing for 10 min at 100 °C. For deposition of perovskite top cell, after starting the spin coating recipe (4000 rpm for 35 s with a ramp of 3 s) 150 μL of HBG perovskite solution was released on the spinning substrate and $N_2$ was blown on the substrate from 20 s till 10 s prior to the end of the spin coating program. Samples were annealed for 30 min at 100 °C. $C_{60}$ (15 nm) and $SnO_x$ (20 nm) were deposited on top. Then, 25 nm ITO was sputtered with an Oxford cluster tool for reference group. For devices with top ZTO, either 75 nm ZTO ($SnO_2$/ZnO = 92 /8 wt%) or 25 nm ZTO ($SnO_2$/ZnO = 99/1 wt%) was sputtered. 300 nm thick silver was thermally evaporated through a busbar mask. Finally, 100 nm $MgF_2$ was thermally evaporated as an antireflection coating layer. Silver and $MgF_2$ evaporations were also conducted with the MBraun evaporation tool.

*Sputtering* Parameters:

*Table S1 The sputtering parameters used for all the TCOs in this work*

| TCO / tool | Power [W] | Pressure [mbar] | $O_2$ flow [sccm] | Ar flow [sccm] |
|---|---|---|---|---|
| Front ITO planar target, DC, static | 40 | $2.6 \times 10^{-3}$ | 0/0.2 | 30 |
| ZTO (92/8) rotary target, DC, in-line | 1500 | $4.3 \times 10^{-3}$ | 18 | 582 |
| ZTO (99/1) rotary target, DC, in-line | 1500 | $4.3 \times 10^{-3}$ | 18 | 582 |
| AZO planar target, DC, in-line | 1000 | $4.3 \times 10^{-3}$ | 2 | 598 |
| Rear ITO rotary target, DC, in-line | 4400 | $3.0 \times 10^{-3}$ | 15 | 285 |

*External quantum efficiency (EQE) measurements*

*EQE* and spectral response (*SR*) were measured in our in-house setup which consists of a Xenon lamp as the light source. The light is chopped at 133 Hz and is directed to a double grating monochromator that produces a single wavelength light. To strengthen the signal and provide bias voltage during measurements, a transimpedance amplifier is connected in series. The signal is finally detected by a lock-in amplifier. Prior to the measurements, the *EQE* response was calibrated with a silicon reference cell. *EQEs* of triple junction devices were measured according to a procedure explained elsewhere [2,3]. In short, for measuring the perovskite top cell, selective

infrared, and red LEDs (920 nm and 740 nm, respectively) were applied to the device. For the middle cell measurement, a combination of blue (460 nm) and infrared LEDs was used and the silicon bottom cell was measured under selective blue and red LEDs. For measurements of each sub cell a bias voltage was applied to the device based on the international standard procedure [3]. The *EQE* was recorded between 300 nm and 1200 nm in 10 nm steps for all three sub-cells. The temperature was kept at 25 °C.

*Current- voltage (IV) measurements*

For triple-junction solar cells, prior to *IV* measurements, the *SR* was measured. *IV* measurements were carried out using an LED solar simulator (Wavelabs Sinus 220). Spectra were calculated according to the procedure described by Chojniak et al. [4] For this procedure only relative *SR* are needed [5]. The measurements were carried out in forward and reverse scan direction. The voltage range was -100 mV to 3200 mV with a step size of 6 mV and a measurement time of 50 s. The temperature was kept at 25 °C.

Single-junction silicon solar cells were measured at a LOANA Solar cell analysis system from PV tools.

*Reflection measurements*

Reflection measurement was done using a LOANA Solar cell analysis system from PV tools.

*UV-Vis measurements*

A Lambda 950 spectrometer from Perkin Elmer equipped with an integrating sphere was used to measure the reflectance (*R*) and transmittance (*T*). Samples were measured in a wavelength range of 250 nm - 1200 nm with a 2 nm step size.

*$iV_{OC}$ imaging*

i$V_{OC}$ images were acquired using a measurement system developed by Fraunhofer ISE and manufactured by Intego GmbH. Originally, the system was equipped with two lasers (450 nm and 808 nm) to allow for photoluminescence (PL) imaging of perovskite silicon dual-junction solar cells. PL imaging of all three subcells was enabled by the incorporation of an additional 700 nm LED. The illumination intensities of the two lasers were adjusted to match the subcell currents generated under the AM1.5g spectrum in the top and bottom cell using the relative *EQE*s following the procedure described by Meusel et al. [5] The 700 nm illumination for the middle cell was set to 1.7 photons $cm^{-2}$ $s^{-1}$. Optical filters were used to separate the luminescence signal from different subcells, before it was captured in a silicon charge-coupled device (CCD) camera. i$V_{OC}$ images were extracted from the PL images calibrating the camera system following the approach described for dual-junction solar cells by Fischer et al. [6]

XPS measurement

XPS spectra were acquired on an EnviroESCA X-ray photoelectron spectrometer from SPECS Surface Nano Analysis GmbH, consisting of a PHOIBOS 150 NAP analyzer, a 1D Delay Line Detector, a source with monochromatic Al Kα radiation at 1486.6 eV, having a beam incident angle of 55° (source to analyzer), and a beam spot size of 100 μm × 200 μm. The survey spectra were obtained with a pass energy of 50 eV and 10 accumulative scans; the high-resolution spectra were obtained at a pass energy of 30 eV and 30 accumulative scans. The energy scale of the instrument was calibrated by using the photoelectron peaks of Au 4f7/2 (83.96 eV) and Ag 3d5/2 (368.50 eV). During data collection the analyzer mode was set to constant pass energy. No binding energy referencing nor charge control were required; the C 1s signals vary in the range of

285.15 eV and 285.23 eV. All samples were measured on the pristine surface and after sputtering 240 s using a GCIB 10S ion gun from Ionoptika (10 kV, $Ar^+$ ion sputtering, 5 × 5 $mm^2$). The data analysis was done with CasaXPS [7]. The following line parameters were used for data fitting:

*Table S2 Parameters used for XPS data fitting*

| Region | Background | Line shape | Component used for quantification | Constrains |
|---|---|---|---|---|
| C 1s | Shirley | LA(1.53,243) | C 1s | same FWHM |
| O 1s | Shirley | GL(40) | O 1s | --- |
| Sn 3d | Spline Tougaard | LA([1.5-1.7],[1.9-2.1],70) | Sn 3d5/2 | same FWHM,<br>Area constraints for d-orbitals<br>DS-Sn 3d = 8.41 eV [8] |
| Zn 2p | Shirley | GL(60) | Zn 2p3/2 | only Zn 2p3/2 fitted |

The semi-quantitative analysis was done considering temperature, mean free pathway and relative sensitivity factors from CasaXPS; an escape depth correction for the correct analyzer angle was applied. The elemental ratios were calculated from the area ratio determined from the peak fitting of the respective orbitals.

*Solar cell modeling*

*FF* losses of single-junction, dual-junction and triple-junction were modelled using the solar cell simulation software Quokka3 [9]. As Quokka3 is limited to single- and dual-junction devices, the multijunction devices are simplified to a single-junction model. For this, the equivalent-circuit solver mode was used which allows to input a $J_0$ value to represent total recombination in the

device. The $J_0$ was adjusted to match typical $V_{OC}$ values, and the generation rate was set to achieve typical $j_{SC}$ values for the different devices, respectively. *FF* losses for a variation of front TCO sheet resistance and finger pitch were then quantified as the *FF* difference to a reference simulation with near-zero sheet resistance (see Table S3).

*Table S3 Solar cell properties to calculate FF losses, reference values for near-zero sheet resistance*

| # junctions | $V_{OC}$ [V] | $j_{SC}$ [mA/cm²] | *FF* [%] | *PCE* [%] |
|---|---|---|---|---|
| 1 | 0.74 | 40.9 | 85.5 | 26.0 |
| 2 | 1.87 | 20.1 | 87.5 | 33.0 |
| 3 | 3.04 | 13.5 | 91.8 | 37.6 |

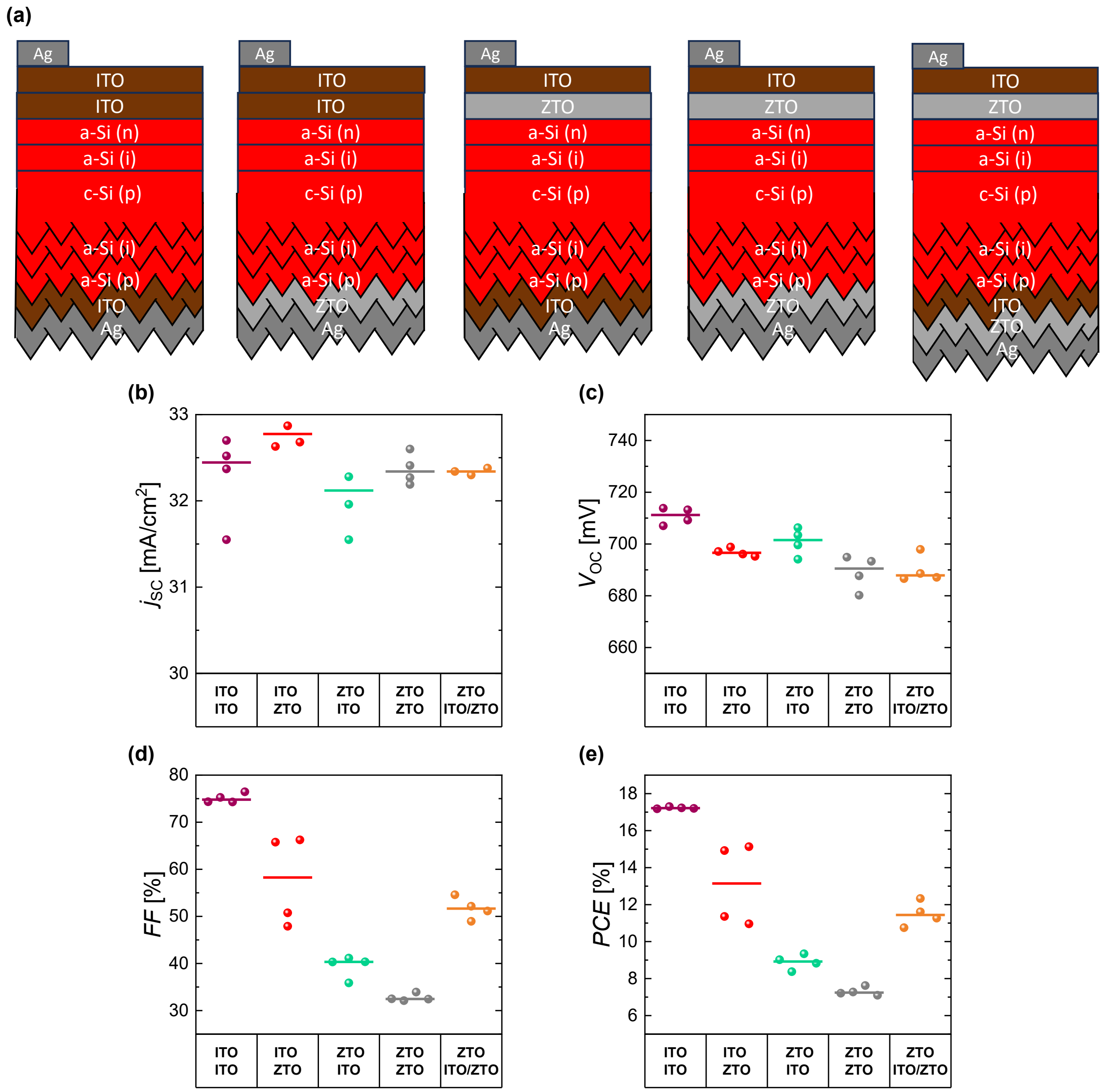


*Figure S1 (a) Schematic and (b-e) the photovoltaic parameters of the silicon heterojunction solar cells. The reference group consists of ITO as front and rear TCO. The rest of the groups are as follows: rear ITO is replaced with ZTO, front ITO is replaced with ZTO, both rear and front ITO are replaced with ZTO, and the last group is with ZTO as front TCO and a bilayer of ITO/ZTO as rear TCO.*

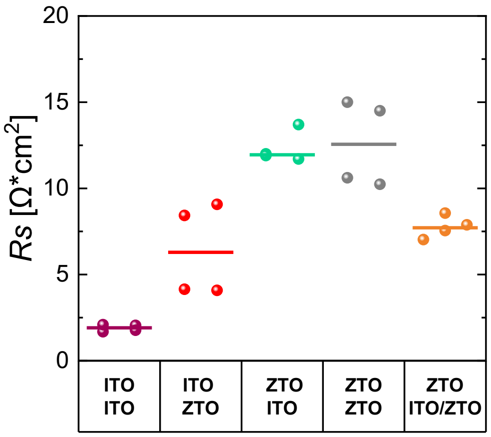


*Figure S2 The series resistance ($R_S$) of the silicon heterojunction solar cells with different rear and front TCOs. The $R_S$ is extracted from the slope of the jV curves at $V_{OC}$ through curve fitting.*

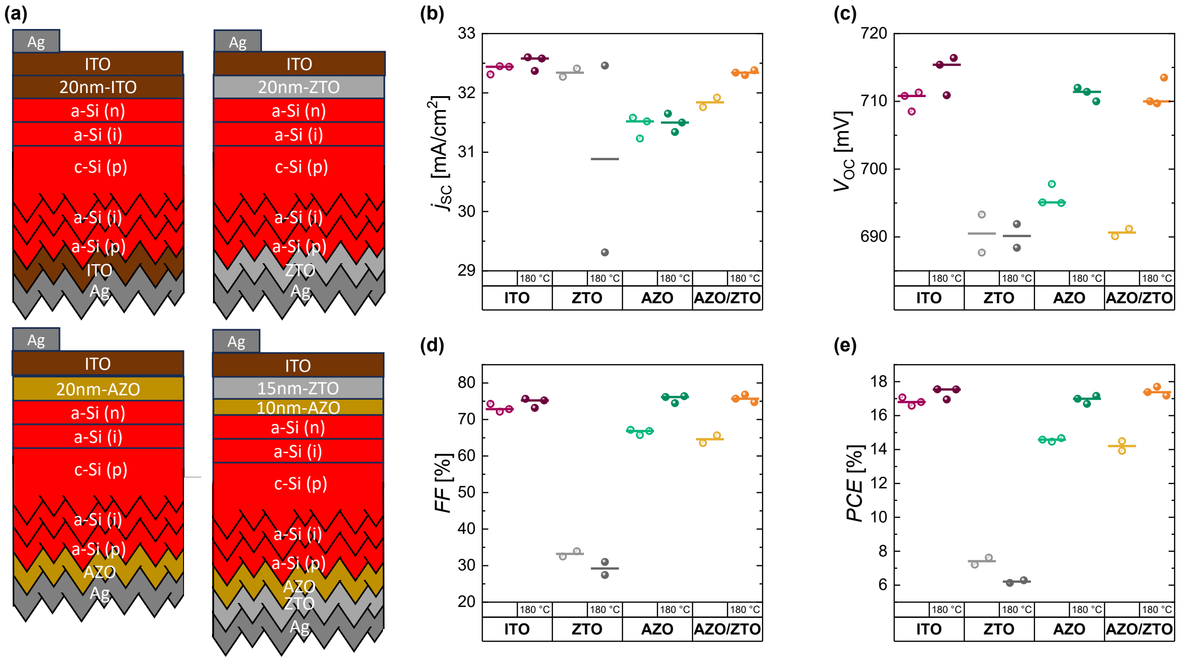


*Figure S3 (a) Schematic and (b-e) the photovoltaic parameters of silicon single-junction devices with ITO, ZTO, AZO and AZO/ZTO as front and rear TCO before and after annealing.*

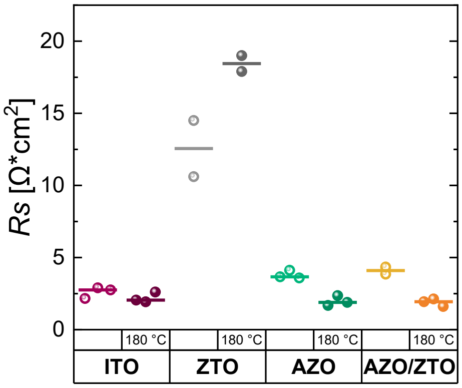


*Figure S4 The series resistance ($R_S$) of the silicon heterojunction solar cells with ITO, ZTO, AZO and AZO/ZTO as front and rear TCO before and after annealing. The $R_S$ is extracted from the slope of the jV curves at $V_{OC}$ through curve fitting.*

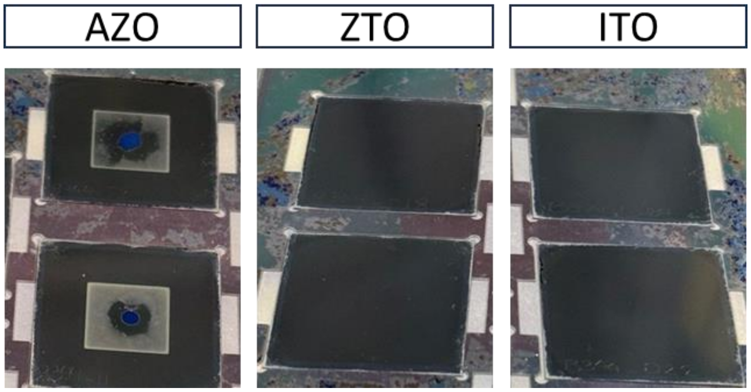


*Figure S5 Perovskite absorber processed on silicon/ITO/PTAA/PFN/MBG perovskite/$C_{60}$/SnOx/(AZO or ZTO or ITO)/2PACz. Note that the ITO, ZTO and AZO are deposited through a shadow mask with 1 $cm^2$ opening. That is the reason of the change in color of perovskite films deposited on AZO only in this area.*

*Table S4 Electrical properties of AZO/ZTO, AZO, ZTO and ITO films. Parameters are extracted from Hall measurements, including Hall mobility (μ), Hall carrier concentration (N *), and sheet resistance (R)*

| Film | Thickness | μ [$cm^2$/Vs] | N *[$\times10^{20}$ $cm^{-3}$] | R [ohm] |
|---|---|---|---|---|
| AZO/ZTO | 10/15 nm | 10.0 | 1.4 | 4400 |
| AZO | 30 nm | 8.9 | 1.3 | 2660 |
| ZTO | 30 nm | 13.8 | 0.4 | 3720 |

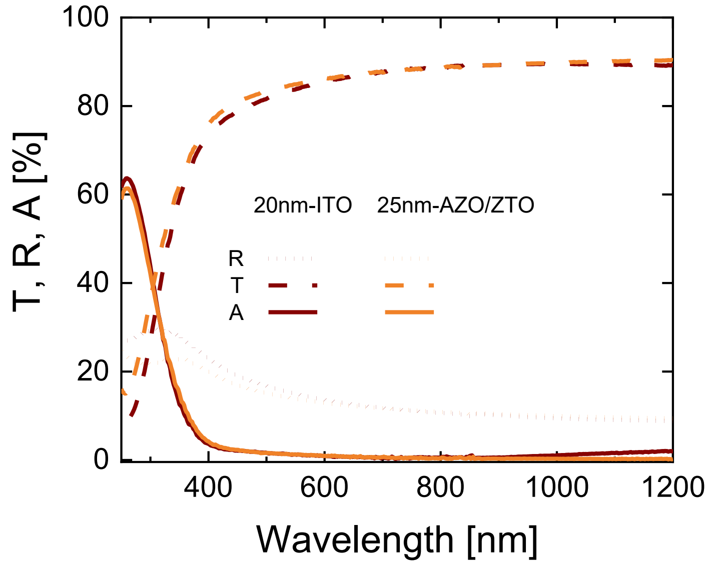


*Figure S6 Transmittance, reflectance and absorptance spectra of ITO and AZO/ZTO layers deposited on glass substrate.*

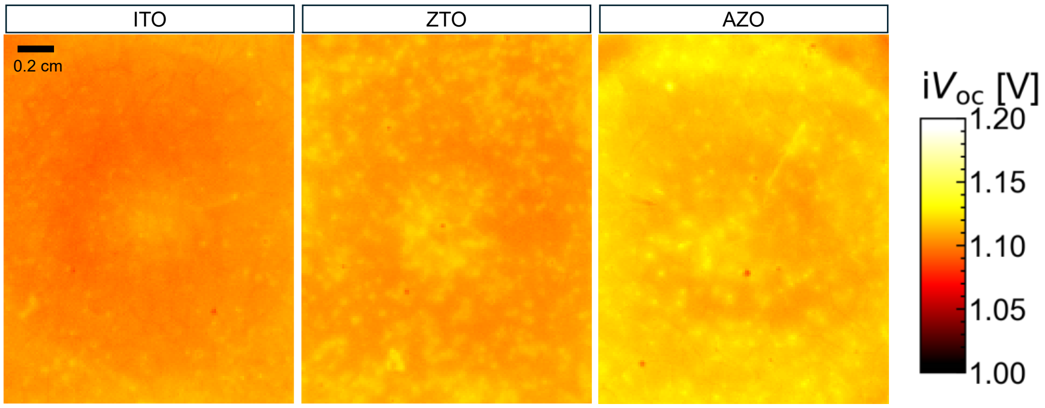


*Figure S7 i$V_{OC}$ images done on glass/perovskite/$C_{60}$/SnOx/TCO stack with different TCO layers to evaluate possible sputter damage. Note that the TCO is sputtered through a shadow mask (1 cm × 1cm) in the center of the sample.*

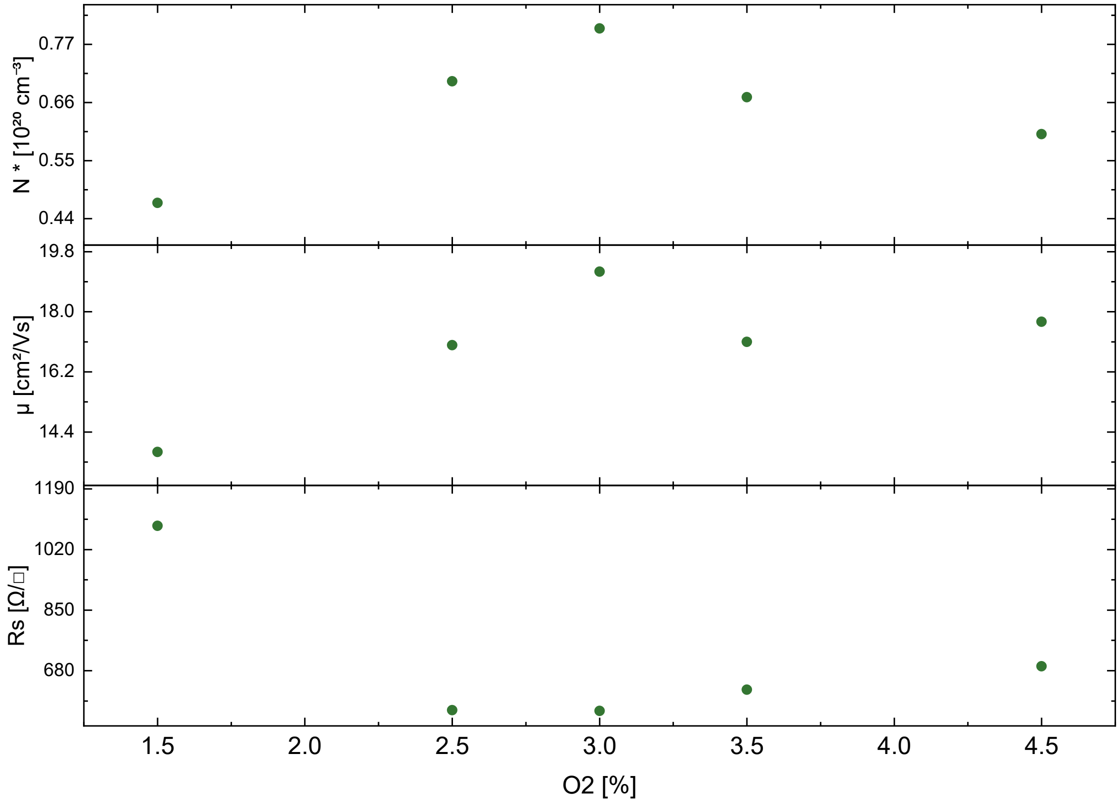


*Figure S8 Electrical properties of ZTO (99/1) films sputtered with different oxygen flow rates. Parameters are extracted from Hall measurements, including Hall mobility (μ), Hall carrier concentration (N *), and mean sheet resistance (Rs)*

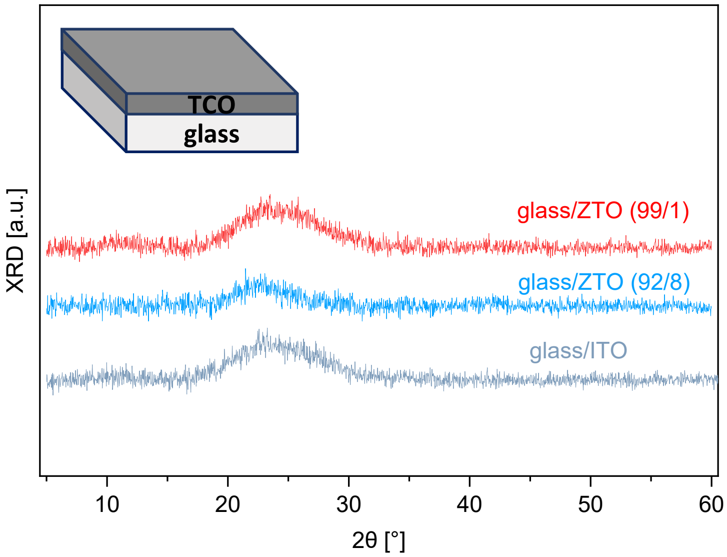


*Figure S9 XRD patterns of ITO and ZTO (92/8) and ZTO (99/1) layers deposited on a glass substrate.*

*Table S5 Elemental composition (Sn, Zn, and O atomic percentages) of ZTO films of different target from XPS measurements. Measurements were conducted on the surface (ET0) and after 240 seconds of sputtering (E240).*

| Species / Target | O 1s | | Sn 3d5/2 | Zn 2p3/2 |
|---|---|---|---|---|
| | O-1 | O-2 | | |
| ZnO 1%-ET0 | 45.8 | 12.3 | 41.6 | 0.3 |
| ZnO 1%-ET240 | 49.0 | 4.1 | 46.6 | 0.3 |
| ZnO 8%-ET0 | 45.7 | 11.9 | 39.0 | 3.4 |
| ZnO 8%-ET240 | 47.9 | 5.3 | 43.1 | 3.7 |

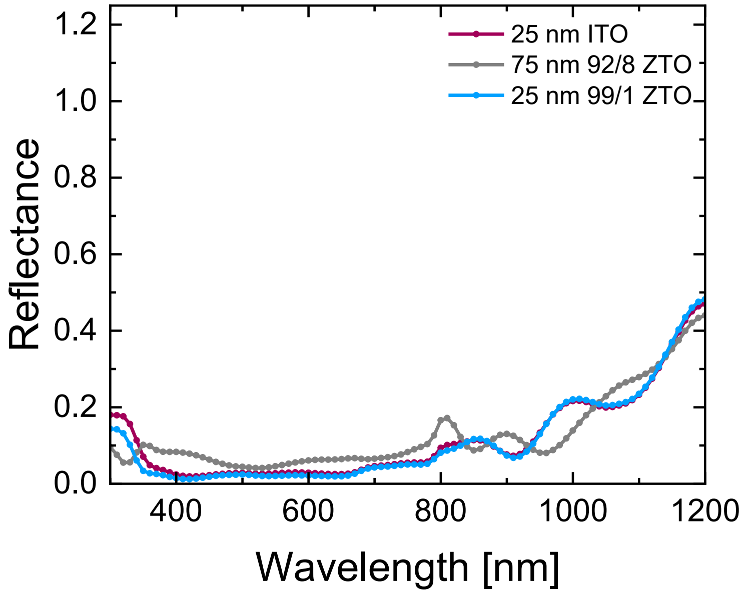


*Figure S10 Reflectance (R) measurement of the triple-junction solar cells with ITO, ZTO (92/8) and ZTO (99/1) as top TCO.*

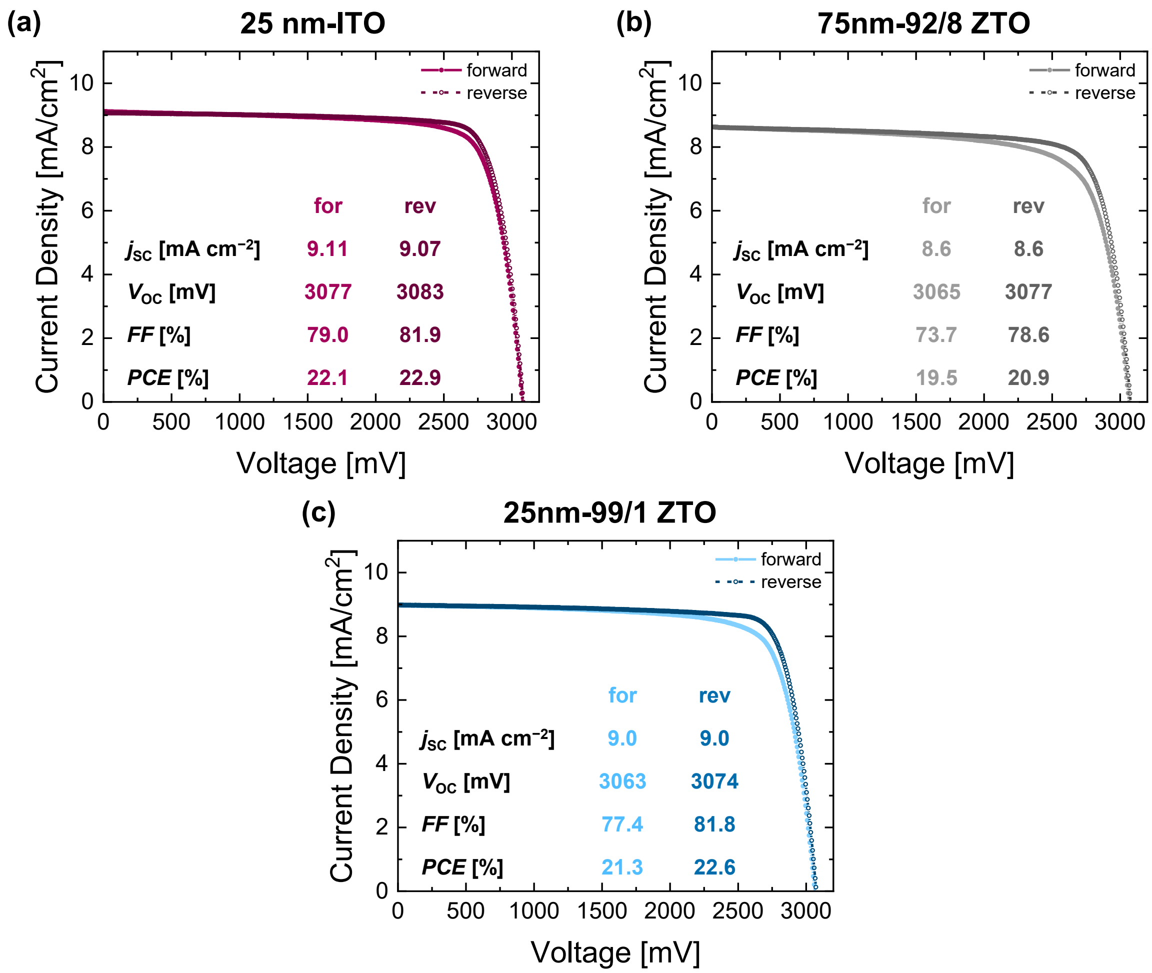


*Figure S11 Champion perovskite/perovskite/silicon triple-junction solar cells with (a) 25 nm ITO, (b) 75 nm ZTO (92/8) and (c) 25 nm ZTO (99/1) as top TCO.*

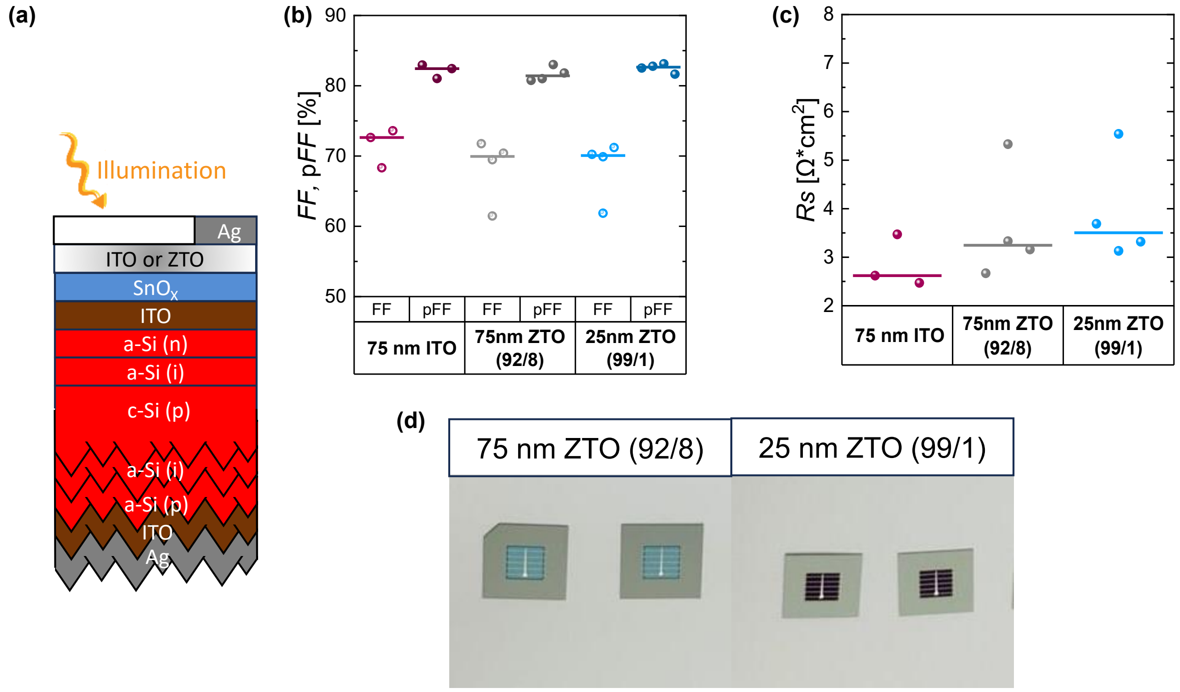


*Figure S12 (a) Schematic of the silicon heterojunction solar cells. The illumination direction is shown with the yellow arrow. (b) The measured FF and pFF and (c) the series resistance ($R_S$) of the silicon heterojunction solar cells with ITO and ZTO as top TCO. The $R_S$ is extracted from the slope of the jV curves at $V_{OC}$ through curve fitting. (d) The images of the silicon solar cells with different ZTO on top.*

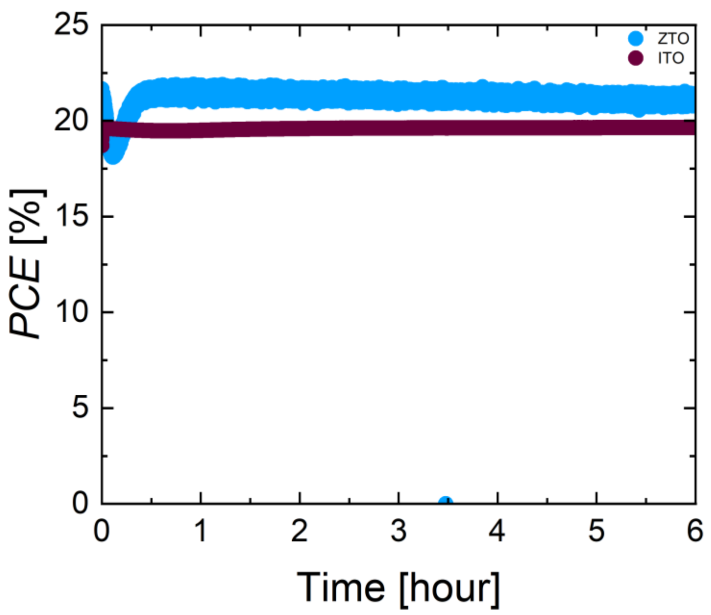


*Figure S13 Efficiency of the indium-free triple-junction solar cell and reference triple-junction solar cell (with ITO based silicon bottom cell and top ITO, and ZTO as recombination layer between perovskite subcells) measured over time at fixed voltage close to maximum power point.*

## References

(1) Heydarian, M.; Shaji, A.; Fischer, O.; Günthel, M.; Karalis, O.; Heydarian, M.; Bett, A. J.; Hempel, H.; Bivour, M.; Schindler, F.*; et al.* Minimizing Open-Circuit Voltage Losses in Perovskite/Perovskite/Silicon Triple-Junction Solar Cell with Optimized Top Cell. *Sol. RRL* **2025**, *9*.

(2) Meusel, M.; Baur, C.; Létay, G.; Bett, A. W.; Warta, W.; Fernandez, E. Spectral response measurements of monolithic GaInP/Ga(In)As/Ge triple-junction solar cells: Measurement artifacts and their explanation. *Progress in Photovoltaics: Research and Applications* **2003**, *11*, 499–514.

(3) IEC; Comission, International ELectrotechnical. *Photovoltaic devices – Part 8-1: Measurement of spectral responsivity of multi-junction photovoltaic (PV) devices*, 1.0th ed., 2017.

(4) Chojniak, D.; Schachtner, M.; Reichmuth, S. K.; Bett, A. J.; Rauer, M.; Hohl-Ebinger, J.; Schmid, A.; Siefer, G.; Glunz, S. W. A precise method for the spectral adjustment of LED and multi-light source solar simulators. *Prog Photovoltaics* **2024**, *32*, 372–389.

(5) Meusel, M.; Adelhelm, R.; Dimroth, F.; Bett, A. W.; Warta, W. Spectral mismatch correction and spectrometric characterization of monolithic III-V multi-junction solar cells. *Progress in Photovoltaics: Research and Applications* **2002**, *10*, 243–255.

(6) Fischer, O.; Bui, A. D.; Schindler, F.; Macdonald, D.; Glunz, S. W.; Nguyen, H. T.; Schubert, M. C. Versatile implied open-circuit voltage imaging method and its application in monolithic tandem solar cells. *Prog Photovoltaics* **2023**, 1–14.

(7) Fairley, N.; Fernandez, V.; Richard-Plouet, M.; Guillot-Deudon, C.; Walton, J.; Smith, E.; Flahaut, D.; Greiner, M.; Biesinger, M.; Tougaard, S.*; et al.* Systematic and collaborative approach to problem solving using X-ray photoelectron spectroscopy. *Applied Surface Science Advances* **2021**, *5*, 100112.

(8) Nyholm, R.; Martensson, N. Core level binding energies for the elements Zr-Te (Z=40-52). *Journal of Physics C: Solid State Physics* **1980**, *13*, L279-L284.

(9) Fell, A.; Schön, J.; Schubert, M. C.; Glunz, S. W. The concept of skins for silicon solar cell modeling. *Solar Energy Materials and Solar Cells* **2017**, *173*, 128–133.
